# Initial therapy affects duration of diarrhoea in critically ill patients with *Clostridioides difficile* infection (CDI)

**DOI:** 10.1186/s13054-019-2648-6

**Published:** 2019-12-09

**Authors:** Carolin F. Manthey, Darja Dranova, Martin Christner, Andreas Drolz, Stefan Kluge, Ansgar W. Lohse, Valentin Fuhrmann

**Affiliations:** 1 First Department of Internal Medicine and Gastroenterology, University Hospital Hamburg-Eppendorf, Martinistr. 52, 20246 Hamburg, Germany; 2 Department of Intensive Care Medicine, University Hospital Hamburg-Eppendorf, Hamburg, Germany; 3 Department of Microbiology, University Hospital Hamburg-Eppendorf, Hamburg, Germany; 4 Medizinische Klinik B für Gastroenterologie und Hepatologie, Universitätsklinikum Münster, Albert-Schweitzer-Campus 1, Gebäude A14, 48149 Münster, Germany

**Keywords:** *Clostridioides difficile* infection (CDI), Intensive care unit (ICU), 28-day mortality, Sepsis, Immunosuppression, Metronidazole, Vancomycin

## Abstract

**Background:**

Critically ill patients in the intensive care unit (ICU) are at high risk for developing *Clostridioides difficile* infections (CDI). Risk factors predicting their mortality or standardized treatment recommendations have not been defined for this cohort. Our goal is to determine outcome and mortality associated risk factors for patients at the ICU with CDI by evaluating clinical characteristics and therapy regimens.

**Methods:**

A retrospective single-centre cohort study. One hundred forty-four patients (0.4%) with CDI-associated diarrhoea were included (total 36.477 patients admitted to 12 ICUs from January 2010 to September 2015). Eight patients without specific antibiotic therapy were excluded, so 132 patients were analysed regarding mortality, associated risk factors and therapy regimens using univariate and multivariate regression.

**Results:**

Twenty-eight-day mortality was high in patients diagnosed with CDI (27.3%) compared to non-infected ICU patients (9%). Patients with non CDI-related sepsis (*n* = 40/132; 30.3%) showed further increase in 28-day mortality (45%; *p* = 0.003). Initially, most patients were treated with a single CDI-specific agent (*n* = 120/132; 90.9%), either metronidazole (orally, 35.6%; or IV, 37.1%) or vancomycin (18.2%), or with a combination of antibiotics (*n* = 12/132; 9.1%). Patients treated with metronidazole IV showed significantly longer duration of diarrhoea > 5 days (*p* = 0.006). In a multivariate regression model, metronidazole IV as initial therapy was an independent risk factor for delayed clinical cure. Immunosuppressants (*p* = 0.007) during ICU stay lead to increased 28-day mortality.

**Conclusion:**

Treatment of CDI with solely metronidazole IV leads to a prolonged disease course in critically ill patients.

## Background

*Clostridioides difficile* infections (CDI) are responsible for most cases of nosocomial infectious diarrhoea in the USA as well as in Europe; mortality rates and hospitalization rates due to CDI are still rising [1, 2]. CDI is acquired through ingestion of the spores of *C. difficile*, mostly in a healthcare setting or through an endogenous source in colonized patients; spores are highly resistant to heat and common decontamination methods [3].

Patients at the intensive care unit (ICU) are highly at risk for nosocomial infections because of immobilization, foreign material and severe comorbidities. In addition, they often receive numerous antibiotics, putting them at further risk for developing CDI [4]. Incidence for CDI in ICU is significantly higher compared to the general hospital population; incidence rates reported vary from 8.7 to 53.9 cases per 10.000 ICU patient days [5, 6]. The total prevalence is estimated between 1 and 2% [7]. The incidence of infections with other gastrointestinal pathogens in hospitalized patients can be neglected [8].

It is generally suggested that mortality rates are increased in critically ill patients who develop CDI [9, 10]. However, some reports show unchanged mortality rates and length of hospital stay in patients at the ICU with CDI [11]. Therefore, a thorough analysis of mortality and risk factors in critically ill patients with CDI is relevant and necessary.

The main risk factor for developing CDI is previous antibiotic therapy which disrupts the patients’ indigenous intestinal flora [12]. Other risk factors in hospitalized patients are low serum albumin levels, older age and severe comorbidities such as decreased renal function [13, 14]. A risk score to predict complicated disease course in the overall hospital population with CDI was developed based on a prospective cohort study. Age (≥ 85 years, OR 4.96; 50–84 years, 1.83), admission due to diarrhoea (OR 3.27), diagnosis at the ICU department (OR 7.03), recent abdominal surgery (OR 0.23) and hypotension (OR 3.25) [15] were identified as independent risk factors. However, there is a lack of data on risk stratification in critically ill patients with CDI.

Current treatment recommendations for CDI rely mainly on the severity of disease [13] and clinical presentations like fever, hypovolemia, lactic acidosis and signs for end-organ failure.

The mainstay of CDI therapy is now considered oral vancomycin whereas metronidazole is reserved for mild disease and intolerance towards vancomycin; furthermore, vancomycin is always recommended in cases with severe disease [16, 17]. Metronidazole IV should be added in severe to fulminant disease [13]. Furthermore, early surgical consultation is recommended in patients who do not respond to conventional therapy within 3 days [13, 18]. Specific recommendations on CDI therapy for critically ill patients are lacking.

Our study objective was the characterization of critically ill patients with CDI and identification of risk factors for unfortunate outcomes as well as analysis of the best antibiotic treatment.

## Patients and methods

### Study population

We performed a retrospective cohort analysis in all critically ill patients diagnosed with CDI in our tertiary care centre to assess risk factors for mortality as primary end point and outcome depending on choice of CDI therapy. In our study population, 2189 patients who developed diarrhoea (total of 36.477 patients admitted to the 12 ICUs of the university hospital in Hamburg, Germany, during the study period (January 2010 until September 2015)) were analysed. The total number of beds in our ICU department is 140; specialties include 5 interdisciplinary ICUs, 1 surgical ICU, 1 internal medicine ICU, 1 neurological and 1 neurosurgical ICU and 3 cardiovascular ICUs.

**Fig. 1:**
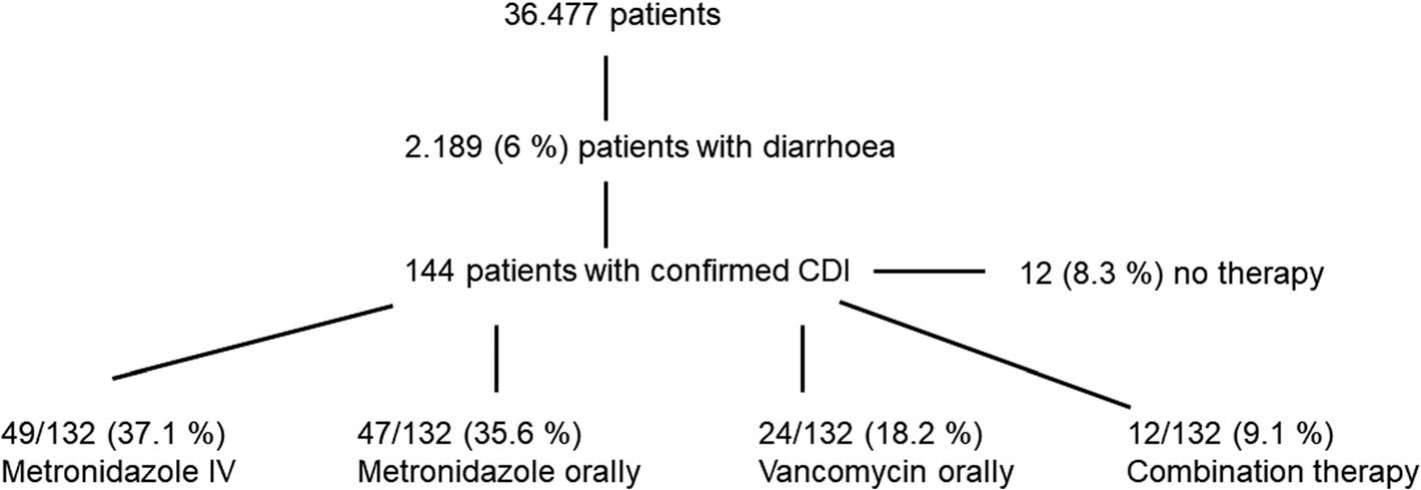
Patients admitted to the ICU between January 2010 and September 2015 with CDI. Legend: shown is initial CDI therapy during first 48 h

Overall, 3188 stool specimens were sent to the microbiology laboratory. Patients tested represented 6.0% of all patients admitted. Ninety-four samples (2.9%) were not processed by the laboratory. *C. difficile* testing was requested in 2209 samples from 1241/36.477 (3.4%) patients and performed as described below. Testing yielded positive results in 242 (glutamate dehydrogenase (GDH) antigen only) and 179 (GDH antigen and toxin A/B; 8.1% of tested samples) samples. In patients negative for GDH antigen determined by enzyme immunoassay (EIA) and positive toxin A/B (EIA), *C. difficile* PCR/*C. difficile* culture was performed. Finally, 144 patients (0.4% of all ICU patients; 6.6% of patients with diarrhoea) were identified as being tested positive for *C. difficile* (EIA for *C. difficile*-GDH and toxin A/B) since some had been tested multiple times. We extracted test results and patient characteristics from our hospital and laboratory information systems and performed individual chart reviews for ICU patients with positive stool cultures. If patients had multiple ICU admissions during one hospital stay, only ICU admissions with CDI diagnosis were included in the analysis. Hundred thirty-two patients out of 144 (91.7%) received specific CDI therapy which was initiated after the positive test result. Twelve patients did not receive a specific CDI therapy on the ICU due to instant death after diagnosis (*n* = 1), decision to limitation of therapy (*n* = 1), lack of symptoms (*n* = 2) or diagnosis after dismissal (*n* = 4). In 4 patients, the reasons for refraining from CDI therapy were unclear (Fig. 1 and Additional file 1: Figure S1). First-line therapy was defined as the CDI-specific antimicrobial treatment for at least 48 h after CDI diagnosis.

This retrospective analysis was performed in accordance to the local regulations of the ethics committee (General Medical Council Hamburg, Ärztekammer Hamburg, reference number WF 11/16).

### Definition of healthcare-associated CDI

CDI was defined as a positive stool test for *C. difficile* GDH and toxin A/B (via EIA) or positive PCR for toxigenic *C. difficile* in combination with a documentation of matching clinical symptoms (diarrhoea, abdominal discomfort). Time point of CDI diagnosis was defined as the date of receiving the positive stool test result.

A severe episode of CDI was defined by fulfilling any one or more of the following criteria at the time point of diagnosis according to literature: serum creatinine concentration > 1.5 mg/dl and > 15,000 white blood cells per μL according to the clinical practice guidelines by the Infectious Diseases Society of America (IDSA) [17].

### Stool testing

For detection of CDI, the C. diff Quick Check Complete EIA (TechLab; Blacksburg, VA, USA) had been used for *C. difficile* glutamate dehydrogenase antigen (GDH) and toxin A/B testing of non-formed stool samples as recommend by the manufacturer. GDH-positive, toxin A/B-negative samples had been retested by Xpert *C. difficile* PCR (Cepheid, Sunnyvale, CA).

### Statistical methods

All continuous variables are reported as median and 25–75% interquartile range (IQR). Categorical variables were compared via chi-square analysis or Fisher’s exact, as appropriate. Metric variables were compared via Mann-Whitney *U* test. Multivariate logistic regression analysis was performed to assess effect of initial medical treatment on duration of diarrhoea > 5 days. Cox regression proportional hazard analysis was performed to assess predictors of mortality. SPSS 24 for Windows (SPSS, Inc., Chicago, IL) was used for statistical analysis. All *p* values reported are two sided, and *p* < 0.05 was considered significant.

## Results

### Patients’ characteristics

The study population was 132 patients treated with CDI-specific antibiotics. Median age of all patients was 70 years (IQR 59–77), and 71.2% were male. 72.7% of the patients needed mechanical ventilation (invasive and non-invasive) during their ICU stay (Table 1); median duration of mechanical ventilation was 11 days (IQR 2–25). 78.8% of the patients received vasopressors for a median of 4 days (IQR 1–12). Renal replacement therapy was necessary in 24.2% of the patients for a median of 9 days (IQR 3–28.5). Main admission diagnoses were sepsis (30.3%) and postoperative admission (29.5%) and cardiac failure (16.7%). In five patients (3.8%), CDI was the leading diagnosis due to severe symptoms; all patients analysed developed CDI during their ICU stay. Detailed patients’ characteristics are illustrated in Table 1.

**Table 1:** Patients’ characteristics in our study population with CDI stratified according to 28-day mortality

Parameter	All patients with CDI *n* = 132	28-day survivors *n* = 96	28-day non-survivors *n* = 36	*p*
Number of patients, *n* (%)	132 (100)	96 (72.2)	36 (27.3)	
Age in years (median, IQR25–75)	70 (59–77)	70.5 (59–75)	70 (59–79)	0.347
Male, n (%)	94 (71.2)	69 (71.8)	25 (69.4)	0.784
Weight (kg) (median, IQR25–75)	75 (65–83)	75 (67–83)	70 (63–81)	0.123
Height (cm) (median, IQR25–75)	172 (165–180)	172 (165–180)	171 (164–176)	0.185
Charlson Comorbidity Index (median, IQR25–75)	5 (3–7)	5 (3–7)	5.5 (4–8)	0.125
SAPS on admission (median, IQR25–75)	41 (33–50)	38.5 (31–48)	44.5 (38–55)	0.003*
TISS28 on admission (median, IQR25–75)	10 (9–17)	14 (9–19)	10 (8–13.5)	0.233
SOFA Score on admission (median, IQR25–75)	6 (4–9)	6.5 (4–9)	6 (4–9)	0.472
SOFA Score on diagnosis (median, IQR25–75)	4 (2–6)	4 (2–6)	6 (4–9)	0.001*
Diagnoses				
Principal diagnosis *C. difficile* infection (CDI), *n* (%)	5 (3.8)	2 (2.1)	3 (8.3)	0.094
Principal diagnosis non CDI-related sepsis, *n* (%)	40 (30.3)	22 (22.9)	18 (50)	0.003*
Principal diagnosis postoperative, *n* (%)	39 (29.5)	31 (32.3)	8 (22.2)	0.259
Principal diagnosis heart failure, *n* (%)	22 (16.7)	16 (16.7)	6 (16.7)	1.000
Principal diagnoses, others, *n* (%)*	36 (27.3)	31 (32.3)	5 (13.9)	0.034*
Neutropenia, *n* (%)	8 (6.1)	3 (3.1)	5 (13.9)	0.021*
Treatment				
Mechanical ventilation overall, *n* (%)	96 (72.7)	66 (68.8)	30 (83.3)	0.094
Vasopressor therapy on admission, *n* (%)	104 (78.8)	72 (75)	32 (88.9)	0.082
Renal replacement therapy (RRT), *n* (%)	32 (24.2)	15 (15.6)	17 (47.2)	< 0.001*
Parenteral nutrition on diagnosis, *n* (%)	27 (20.5)	15 (15.6)	12 (33.3)	0.025*
Enteral nutrition on diagnosis, *n* (%)	125 (94.7)	93 (96.9)	32 (88.9)	0.068
Outcome				
ICU stay (days) (median, IQR25–75)	14 (6–29)	13.5 (6–28)	14 (8–35)	0.688
Hospital stay (median, IQR25–75)	37.5 (18–61)	39.5 (23–62.5)	24.5 (15–54)	0.091
Medication				
Proton pump inhibitors, *n* (%)	126 (95.5)	91 (94.8)	35 (97.2)	0.550
Immunosuppressants, *n* (%)	33 (25)	18 (18.8)	15 (41.7)	0.007*
Steroids > 10 mg/day, *n* (%)	28 (21.2)	14 (14.6)	14 (38.9)	0.002*
Calcineurin inhibitors, *n* (%)	12 (9.1)	6 (6.3)	6 (16.7)	0.064
Mycophenolic acid, *n* (%)	4 (3)	2 (2.1)	2 (5.6)	0.300
Azathioprine (AZA), *n* (%)	4 (3)	2 (2.1)	2 (5.6)	0.300

*In some patients, > 1 diagnosis was encoded as principal diagnosis

Median stay on ICU was 14 days (IQR 6–29); overall median hospital stay was 37.5 days (IQR 18–61).

Most patients received proton pump inhibitors (PPI) during ICU stay (95.5%). Furthermore, 25% of all patients were given immunosuppressive drugs, mostly steroids in a dosage > 10 mg/day (21.2%). Other immunosuppressive medications included calcineurin inhibitors (9.1%), mycophenolic acid (3%) and azathioprine (3%).

The median time between ICU admission and diagnosis of CDI was 13.5 days (IQR 5–28) (Table 2). 18.9% (*n* = 25) of all patients suffered from recurrent CDI. Severe CDI was present in 60.6% of all patients. Toxic megacolon occurred in one patient. Overall, two patients (1.5%) required colectomy due to fulminant CDI.

**Table 2:** CDI-specific patients’ characteristics of the study population stratified for 28-day mortality

Patients’ characteristics	All patients with CDI *n* = 132	28-day survivors *n* = 96	28-day non-survivors *n* = 36	*p*
Time from ICU admission to CDI diagnosis (days) (median, IQR 25–75)	13.5 (5–28)	12.5 (5–27)	17.5 (5–34)	0.378
Antibiotic therapy on ICU, *n* (%)	126 (95.5)	91 (94.8)	35 (97.2)	0.550
Recurrent CDI, *n* (%)	25 (18.9)	16 (16.7)	9 (25)	0.276
Severe CDI, *n* (%)	80 (60.6)	55 (57.3)	25 (69.4)	0.349

Data on antibiotic therapy before admission were incomplete. While on ICU, 95.5% (*n* = 126) of patients with CDI were additionally treated with non-CDI antimicrobial therapies. Patients received a median number of 3 (IQR 1–5) different antibiotics and were treated for a median of 10 days (IQR 5–24). Carbapenems were prescribed in 64.4%, acylaminopenicillins in 40.9%, vancomycin IV in 40.2%, cephalosporins in 38.6% and fluoroquinolones in 40.2% of all patients who received antibiotics.

### CDI-specific therapy

First-line therapy within 48 h after diagnosis was metronidazole IV in 37.1% (*n* = 49) of all patients, 35.6% (*n* = 47) received metronidazole orally and 18.2% (*n* = 24) were given vancomycin orally. Only 9.1% were treated with a combination therapy (*n* = 12) initially (Fig. 1).

Severity of CDI did not influence first-line therapy in these patients since equal parts in both patient groups with severe (*n* = 80, 60.6%) and non-severe CDI (*n* = 52, 39.4%) were treated with metronidazole IV (37.7 vs. 36.5%), metronidazole orally (33.8 vs. 38.5%), vancomycin orally (20 vs. 15.4%) and combination therapy (8.8 vs. 9.6%) (Additional file 1: Table S1). After 2014, treatment choices started to change; here only 18.9% (*n* = 7/34) of patients with severe CDI received metronidazole IV.

A detailed overview of all CDI medications over time is illustrated in Additional file 1: Figure S1. Briefly, 62.1% (*n* = 82/132) received only one substance (metronidazole or vancomycin), whereas 37.9% (*n* = 50/132) received a combination of drugs, mostly metronidazole IV plus vancomycin orally (15.9%, *n* = 21/132).

Therapy was adjusted over the course of disease: most patients obtained metronidazole intravenously (IV) (53.8%, *n* = 71); 45.5% (*n* = 60) were treated with metronidazole orally while 43.2% (*n* = 57) received vancomycin orally. Only 2 patients (1.5%) obtained vancomycin per rectum (VPR). Two patients were additionally treated with teicoplanin or fidaxomicin orally, respectively (Additional file 1: Figure S1). Median length of CDI therapy was 8 days (IQR 4–11 days).

**Fig. 2:**
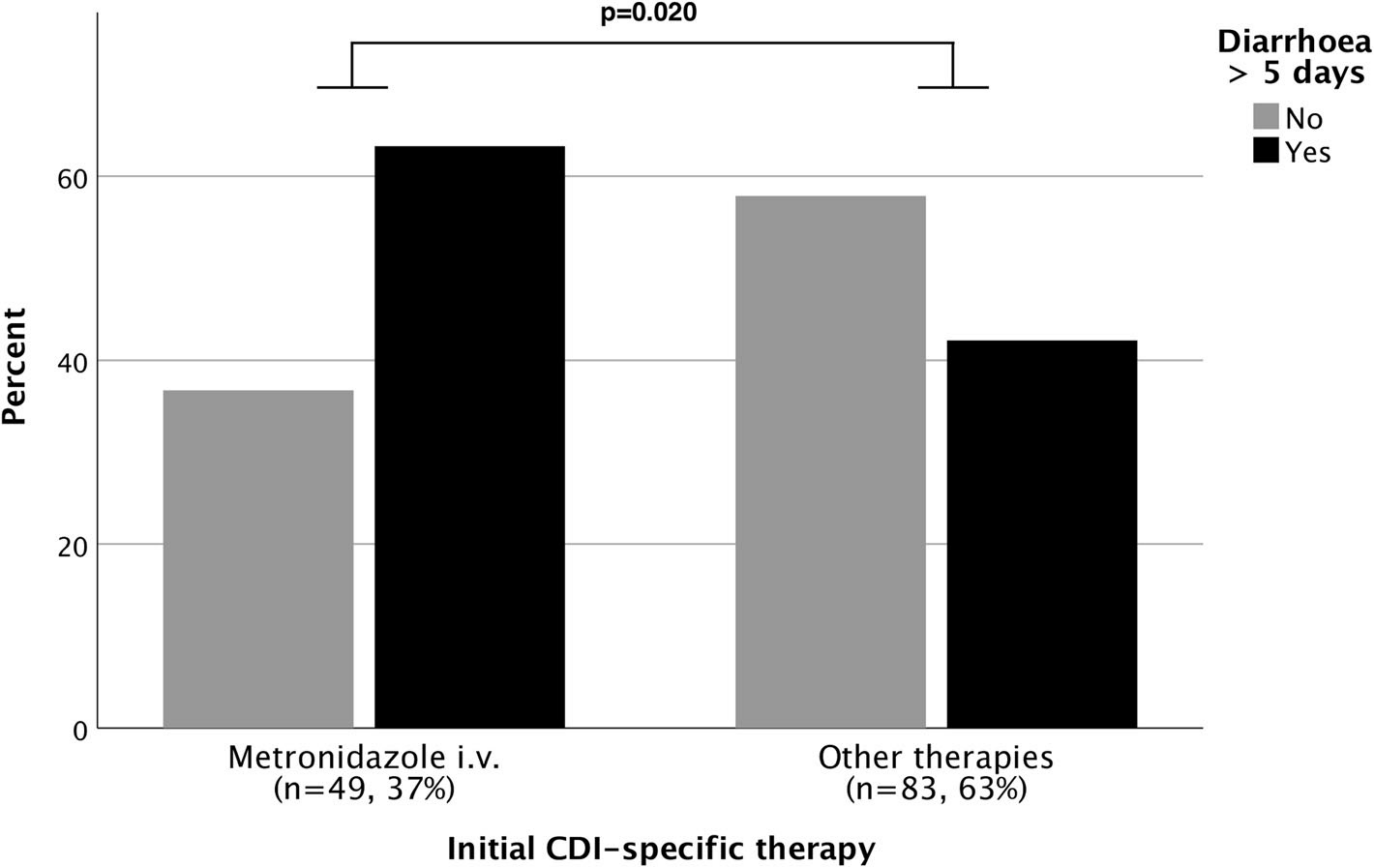
Length of diarrhoea in relation to initial CDI therapy

Median length of diarrhoea was 5 days (IQR3–8). Half of the patients were suffering from prolonged diarrhoea > 5 days (*n* = 66, 50%, see Additional file 1: Table S3). First-line metronidazole IV was the only CDI-specific therapy associated significantly with prolonged diarrhoea > 5 days (*p* = 0.020) as shown in Fig. 2. Furthermore, metronidazole IV as initial therapy was associated with prolonged diarrhoea independently of gender, age, severity of CDI, SAPS on diagnosis and presence of sepsis (OR 2.499, 95% CI 1.150–5.431, *p* = 0.021) as illustrated in Table 3. Patients treated with metronidazole IV exhibited increased 28-day (34.7 vs. 27.7%, *p* = 0.141) and 90-day mortality (40.8 vs. 26.5%, *p* = 0.088) compared to patients treated with a different regime, although not statistically significant. Increased mortality in this patient subgroup rather shows the overall increased morbidity also represented by higher rates of renal replacement therapy (32.7 vs. 19.3%, *p* = 0.083) and more frequent presence of sepsis (34.7 vs. 27.7%, *p* = 0.399) (Additional file 1: Table S3).

**Table 3:** Multivariable logistic regression analysis of metronidazole (initial therapy) as predictor for increased length of diarrhoea

Parameters	OR (95% CI)	*p*
Metronidazole IV first 48 h	2.362 (1.143–4.882)	0.020*
Metronidazole IV (male gender)*	2.459 (1.176–5.141)	0.017*
Metronidazole IV (male gender/age)*	2.324 (1.104–4.892)	0.026*
Metronidazole IV (male gender/age/severe CDI)*	2.333 (1.102–4.940)	0.027*
Metronidazole IV (male gender/age/severe CDI/SAPS on diagnosis)*	2.397 (1.122–5.121)	0.024*
Metronidazole IV (male gender/age/severe CDI/SAPS on diagnosis/sepsis)*	2.499 (1.150–5.431)	0.021*

*Corrected for covariates in brackets

### Mortality and associated risk factors

Twenty-eight- and 90-day mortality rates were 27.3% and 31.8%, respectively. Apart from severity of illness, impaired kidney and liver function, presence of sepsis not related to CDI, neutropenia and necessity of parenteral nutrition were associated with significantly higher mortality rates as illustrated in Table 1 and Additional file 1: Table S2. Patients receiving immunosuppressants (*p* = 0.007), especially steroids > 10 mg per day (*p* = 0.002) during their ICU stay, exhibited increased 28-day mortality as illustrated in Table 1. We observed a trend of higher mortality in patients treated with metronidazole IV initially as single-agent therapy (28-day mortality 34.7% and 90-day mortality 40.8%) (Additional file 1: Table S4); otherwise, none of the employed CDI-therapy regimens had a relevant impact on mortality. Furthermore, both patients who received vancomycin enemas died within 90 days due to severe course of disease.

Multivariate Cox regression analysis identified presence of immunosuppressive therapy (HR 2.118, 95% CI 1.068–4.198, *p* = 0.032) and severity of illness represented by SOFA score (HR 1.216, 95% CI 1.109–1.334, *p* = < 0.001) as independent predictors of 28-day mortality.

## Discussion

CDI is the most common cause of nosocomial infectious diarrhoea in Western hospitals [19]. However, there is a lack of data regarding its clinical impact in critically ill patients and the consequences of different therapeutic approaches on cure of disease and survival. Therefore, we assessed the prevalence of CDI in a large cohort of critically ill patients, identified factors influencing the duration and course of the disease and evaluated predictive factors regarding survival in critically ill patients with CDI.

The prevalence of CDI in our large cohort of critically ill patients was lower compared to previous reports (0.4% vs. 2%) [7]. In our cohort, ICU mortality in CDI patients was significantly higher than in critically ill patients without CDI (20.5% versus 9%, *p* < 0.05) confirming previous data [7, 20]. Independent predictors of 28-day mortality were high SOFA score and immunosuppressive therapy in patients with CDI.

The use of proton pump inhibitors before or during ICU stay had no impact on outcome in our cohort of critically ill patients suffering from CDI. This is in accordance to previous reports and may probably be the consequence of the fact that a high proportion of ICU patients receive PPIs making it difficult to detect a distinct effect [21]. Previous studies showed varying incidence of CDI in hospitalized patients under the influence of PPIs. However, the long-term use of PPIs (> 2 days) seems to be an independent risk factor of CDI development in critically ill patients [22]. Therefore, further prospective studies are warranted assessing risks of CDI development and side effects of PPI in this heterogeneous group of patients.

The use of glucocorticoids is associated with increased mortality in patients with CDI in the general hospital population [23]. In our study, we also observed increased mortality rates in patients receiving glucocorticoids independently of severity and type of underlying disease. Necessity of glucocorticoid therapy should be critically evaluated in critically ill patients with CDI.

Although it has been reported that enteral tube feeding poses a risk factor for CDI [24], our data identified parenteral nutrition as risk factor for increased 28-day mortality in critically ill patients with CDI. Apart from the level of sickness in terms of choice of nutrition therapy in critically ill patients, our data supports the recommendation of the European Society of Intensive Care Medicine, not to delay early enteral nutrition in critically ill patients with infections.

Accordingly, critically ill patients’ primarily enteral nutrition seems to be vital, as it is also the general recommendation of nutrition in critical illness [25, 26].

Choice of CDI therapy has been reported to influence mortality rate and should therefore be determined appropriately [27, 28]. Although there are several recommendations on treatment of CDI based on severity of disease, specific recommendations for CDI therapy in patients at the ICU are lacking [13].

In our cohort, more than 90% of CDI infections were treated by a monotherapy and only 9.1% of all patients received a combination therapy in the beginning. Most patients were treated with metronidazole orally or IV; this treatment is currently only recommended in mild disease in patients who do not tolerate vancomycin [17]. Since our data collection starts in 2010, therapy recommendations were still relying on metronidazole as first-line treatment. In the following years, studies showed the superiority of vancomycin in treating patients with severe CDI [27].

Interestingly, we observed a significantly decreased clinical success rate in patients treated with metronidazole IV as first-line therapy. Critically ill patients frequently suffer from impaired intestinal transport, and oral drug application might be challenging. Consequently, intravenous treatments are often used. Intravenous metronidazole is partly secreted into the gut lumen in the inflamed colon but concentrations may vary and might not suffice to effectively treat CDI [29]. Now, our data suggest that oral therapy should be included in CDI treatment regimens as far as possible, either vancomycin or metronidazole.

Addition of intravenous metronidazole to vancomycin has been shown to decrease mortality rates in critically ill patients in a retrospective study [28]. However, we could not observe a systematic advantage of combination therapy in this small subgroup (*n* = 12) in our study.

There is certainly a limitation in our analysis due to the retrospective setting, low number of CDI cases at our hospital and therefore lack of opportunity for propensity score matching. However, to our knowledge, this study is one of the largest studies on incidence and treatment of CDI, especially in the ICU setting.

## Conclusion

CDI further harms critically ill patients by increasing 28-day mortality and in case of prolonged diarrhoea the length of their hospital stay. Appropriate therapy should be initiated promptly to shorten duration of diarrhoea. Our data point to the fact that metronidazole IV as a single agent might not represent an efficient initial monotherapy. Future studies should address the evaluation of combination therapy in critically ill patients with CDI. Since immunosuppressive therapy was identified as an independent predictor of increased 28-day mortality, patients should be carefully evaluated whether immunosuppressive therapy is indicated in case of concurrent CDI.

## Supplementary information

**Additional file 1: Figure S1.** CDI specific therapy in all ICU patients with CDI 2010–2015. Legend: Abbreviations: IV: intravenously, VPR: Vancomycin per rectum. **Table S1.** Treatment strategy according to CDI severity during first 48 h after diagnosis of CDI. Legend: A: before 2014, B: 2014-October 2015. **Table S2.** Characteristics of all treated patients with CDI. Legend: Univariate regression analysis of risk factors of 28-day survivors vs. 28-day-nonsurvivors. Abbreviations: BUN: blood urea nitrogen, CRP: C-reactive protein, vol.: volume, MAP: mean arterial pressure. **Table S3.** Patients’ characteristics of the study population with CDI stratified according to diarrhoea 5 days. **Table S4.** Patients’ characteristics of the study population with CDI stratified according to initial CDI therapy (first 48 h).

## Data Availability

The datasets used and/or analysed during the current study are available from the corresponding author on reasonable request.
