# Supplementary material for: Initial therapy affects duration of diarrhoea in critically ill patients with Clostridioides difficile infection (CDI)
Source: Crit Care. 2019 Dec 9;23:399. doi: 10.1186/s13054-019-2648-6 (PMC6902451; doi:10.1186/s13054-019-2648-6)
Supplement: Supplementary file 1 — Additional file 1: Figure S1. CDI specific therapy in all ICU patients with CDI 2010–2015. Legend: Abbreviations: IV: intravenously, VPR: Vancomycin per rectum. Table S1. Treatment strategy according to CDI severity during first 48 h after diagnosis of CDI. Legend: A: before 2014, B: 2014-October 2015. Table S2. Characteristics of all treated patients with CDI. Legend: Univariate regression analysis of risk factors of 28-day survivors vs. 28-day-nonsurvivors. Abbreviations: BUN: blood urea nitrogen, CRP: C-reactive protein, vol.: volume, MAP: mean arterial pressure. Table S3. Patients’ characteristics of the study population with CDI stratified according to diarrhoea 5 days. Table S4. Patients’ characteristics of the study population with CDI stratified according to initial CDI therapy (first 48 h). [file 13054_2019_2648_MOESM1_ESM.pdf]

## Supporting information

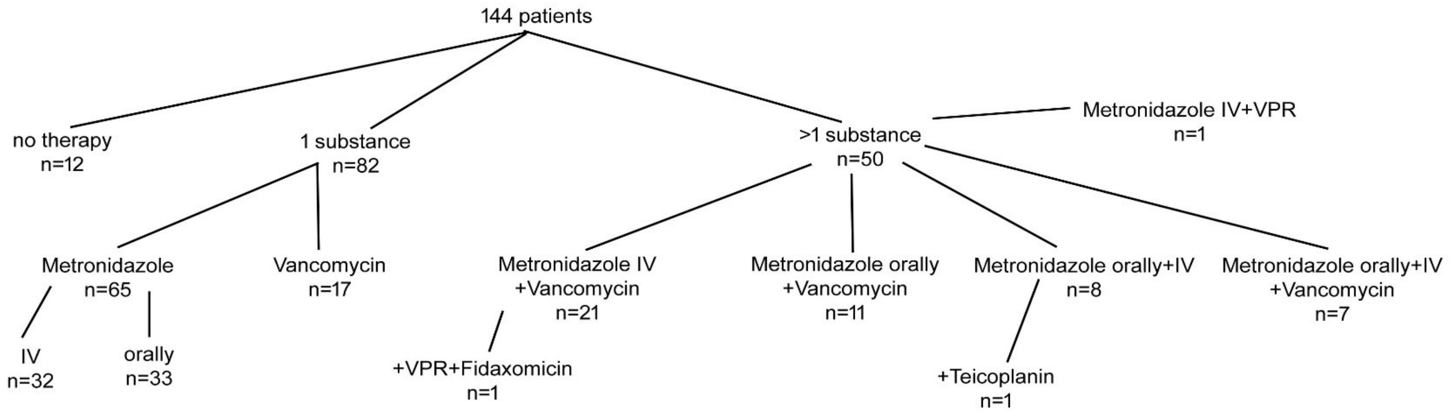


**Figure S1:** CDI specific therapy in all ICU patients with CDI 2010-2015. Legend: Abbreviations: IV: intravenously, VPR: Vancomycin per rectum

**Table S1:** Treatment strategy according to CDI severity during first 48 h after diagnosis of CDI.

| **A: Medication during first 48 h after diagnosis** | **All patients with CDI n=132** | **Severe CDI n=80** | **Non-severe CDI n=52** |
|---|---|---|---|
| Initial Combination therapy | 12 (9.1) | 7 (8.8) | 5 (9.6) |
| Initial metronidazole IV single | 49 (37.1) | 30 (37.5) | 19 (36.5) |
| Initial vancomycin orally single | 24 (18.2) | 16 (20) | 8 (15.4) |
| Initial metronidazole orally single | 47 (35.6) | 27 (33.8) | 20 (38.5) |
| **B: Medication during first 48h after diagnosis (2014-2015)** | **All patients with CDI n=54** | **Severe CDI n=34** | **Non-severe CDI n=20** |
| Initial Combination therapy | 9 (15.5) | 5 (13.5) | 4 (20) |
| Initial metronidazole IV single | 11 (19) | 7 (18.9) | 4 (20) |
| Initial vancomycin orally single | 13 (22.4) | 9 (24.3) | 4 (20) |
| Initial metronidazole orally single | 21 (36.2) | 13 (35.1) | 8 (40) |

Legend: A: before 2014, B: 2014-October 2015.

**Table S2:** Characteristics of all treated patients with CDI.

| | **All patients with CDI n=132** | | **28-day-survivors n=96** | | **28-day-non-survivors n=36** | | **p** | |
|---|---|---|---|---|---|---|---|---|
| **Parameters** | **admission** | **diagnosis** | **admission** | **diagnosis** | **admission** | **diagnosis** | **admission** | **diagnosis** |
| **Laboratory characteristics** | | | | | | | | |
| Thrombocytes (Median, IQR25-75) | 197 (116-282) | 249 (128-385) | 198 (128-292) | 262 (164-394) | 172 (87-212) | 143 (71-318) | **0.007*** | **0.006*** |
| Leukocytes (Median, IQR25-75) | 11.5 (7-17) | 12.2 (7.9-17,7) | 11.5 (7.9-17) | 12.5 (9.1-17,9) | 11.5 (3.8-17,5) | 12.2 (3.7-17.6) | 0.655 | 0.482 |
| Hemoglobin (Median, IQR25-75) | 9.5 (8.7-10.9) | 8.8 (8-9.9) | 9.8 (8.9-11) | 8.8 (8-9.9) | 9.7 (9-11.6) | 9.3 (8-10.5) | 0.489 | 0.206 |
| BUN (Median, IQR25-75) | 23 (14-37.5) | 27 (17-40) | 22 (13-38) | 26 (17-39) | 29 (21-44) | 32 (23-41) | **0.002*** | **0.049*** |
| Creatinine (Median, IQR25-75) | 1.3 (0.9-2.1) | 1.1 (0.8-2) | 1.3 (0.9-2.1) | 1.1 (0.7-2) | 1.6 (1.1-2.6) | 1.3 (0.9-2.4) | **0.032*** | 0.067 |
| CRP (Median, IQR25-75) | 93 (24-150) | 108 (52-157) | 82 (20-159) | 108 (58-149) | 104 (63-148) | 104 (44-163) | 0.051 | 0.894 |
| Lactate (Median, IQR25-75) | 1.2 (0.8-2) | 1 (0.7-1.4) | 1.2 (0.9-1.8) | 0.9 (0.7-1.2) | 1.4 (0.8-2.5) | 1.3 (0.8-1.9) | 0.450 | **0.008*** |
| Albumin (Median, IQR25-75) | 19 (17-23) | 17 (15-22) | 19 (17-23) | 18 (15-22) | 17 (16-19) | 17 (15-22) | 0.127 | 0.844 |
| Bilirubin (Median, IQR25-75) | 0.6 (0.3-1) | 0.4 (0.2-0.7) | 0.6 (0.3-0.9) | 0.4 (0.2-0.6) | 0.9 (0.5-1.9) | 0.7 (0.4-1.6) | **0.001*** | **<0.001*** |
| **Clinical characteristics** | | | | | | | | |
| Urine vol. (l), (Median, IQR25-75) | 1350 (658-2300) | 1665 (710-2488) | 1515 (748-2501) | 1910 (1317-2862) | 915 (460-1535) | 950 (173-1585) | **0.006*** | **<0.001*** |
| SAPS (Median, IQR25-75) | 41 (33-50) | 36.5 (29-45) | 38.5 (31-48) | 34 (28-43) | 44.5 (38-55) | 43 (34-55) | **0.003*** | **0.001*** |
| TISS28 (Median, IQR25-75) | 10 (9-17) | 10 (5-14) | 14 (9-19) | 10 (5-14) | 10 (8-14) | 10 (5-13) | 0.233 | 0.907 |
| SOFA Score (Median, IQR25-75) | 6 (4-9) | 4 (2-6.3) | 6.5 (4-9) | 4 (2-6) | 6 (4-9) | 6 (4-9) | 0.472 | **<0.001*** |
| Temp. (C°) (Median, IQR25-75) | 36.7 (36-37.3) | 37.1 (36.7-37.6) | 36.7 (36.1-37.3) | 37.2 (36.7-37.6) | 36.7 (35.8-37.3) | 37.1 (36.4-37.7) | 0.698 | 0.442 |

| Therapy/Treatment | | | | | | | | |
|---|---|---|---|---|---|---|---|---|
| Parenteral nutrition, n (%) | 23 (17.4) | 27 (20.5) | 15 (15.6) | 15 (15.6) | 8 (22.2) | 12 (33.3) | 0.374 | **0.025*** |
| Enteral nutrition, n (%) | 105 (79.5) | 125 (94.7) | 79 (82.3) | 93 (96.9) | 26 (72.2) | 32 (88.9) | 0.201 | 0.068 |

Legend: Univariate regression analysis of risk factors of 28-day survivors vs. 28-day-nonsurvivors. Abbreviations: BUN: blood urea nitrogen, CRP: C-reactive protein, vol.: volume, MAP: mean arterial pressure

**Table S3:** Patients` characteristics of the study population with CDI stratified according to diarrhoea > 5 days.

| Parameter | All patients n=132 | Diarrhoea < 5 days n=66 | Diarrhoea > 5 days n=66 | p |
|---|---|---|---|---|
| Severe CDI, n (%) | 87 (65.9) | 38 (57.6) | 49 (74.2) | 0.098 |
| Male, n (%) | 94 (71.2) | 45 (68.2) | 49 (74.2) | 0.701 |
| Recurrent CDI, n (%) | 25 (18.9) | 12 (18.2) | 13 (19.7) | 0.505 |
| Principal diagnosis sepsis, n (%) | 40 (30.3) | 24 (36.4) | 16 (24.2) | 0.130 |
| Neutropenia, n (%) | 8 (6.1) | 4 (6.1) | 4 (6.1) | 1.000 |
| **Treatment** | | | | |
| Parenteral nutrition on diagnosis, n (%) | 27 (20.5) | 15 (22.7) | 12 (18.2) | 0.281 |
| Enteral nutrition on diagnosis, n (%) | 125 (94.7) | 62 (93.9) | 63 (95.5) | 0.698 |
| Proton pump inhibitors, n (%) | 126 (95.5) | 63 (95.5) | 63 (95.5) | 1.000 |
| Immunosuppressants, n (%) | 33 (25) | 15 (22.7) | 18 (27.3) | 0.546 |

**Table S4:** Patients` characteristics of the study population with CDI stratified according to initial CDI therapy (first 48 h)

| **Parameter** | **All patients with CDI n=132** | **Metronidazole IV n=49** | **Other CDI therapy n=83** | **p** |
|---|---|---|---|---|
| Number of patients, n (%) | 132 (100) | 49 (37.1) | 83 (62.9) | |
| Age in years (Median, IQR25-75) | 70 (59-77) | 67 (58-74) | 72 (60-78) | 0.093 |
| Male, n (%) | 94 (71.2) | 31 (63.3) | 63 (75.9) | 0.121 |
| Charlson Comorbidity Index (Median, IQR25-75) | 5 (3-7) | 4 (2-7) | 5 (3-7) | 0.136 |
| SAPS on diagnosis (Median, IQR25-75) | 37 (29-45) | 40 (30-46) | 34 (29-45) | 0.167 |
| SOFA Score on admission (Median, IQR25-75) | 6 (4-9) | 6 (5-10) | 6 (4-8) | 0.105 |
| SOFA Score on diagnosis (Median, IQR25-75) | 4 (2-6) | 5 (3-6) | 4 (2-7) | 0.350 |
| **Diagnoses** | | | | |
| Principal diagnosis *C. difficile* infection (CDI), n (%) | 5 (3.8) | 2 (4.1) | 3 (3.6) | 0.892 |
| Principal diagnosis Sepsis, n (%) | 40 (30.3) | 17 (34.7) | 23 (27.7) | 0.399 |
| Principal diagnosis Postoperative, n (%) | 39 (29.5) | 17 (34.7) | 22 (26.5) | 0.319 |
| Neutropenia, n (%) | 8 (6.1) | 4 (8.2) | 4 (4.8) | 0.437 |
| Severe CDI, n (%) | 87 (65.9) | 33 (67.3) | 54 (65.1) | 0.789 |
| **Treatment** | | | | |
| Mechanical ventilation overall, n (%) | 96 (72.7) | 37 (75.5) | 59 (71.1) | 0.488 |
| Vasopressor therapy on admission, n (%) | 104 (78.8) | 40 (81.6) | 64 (77.1) | 0.539 |
| Renal replacement therapy (RRT), n (%) | 32 (24.2) | 16 (32.7) | 16 (19.3) | 0.083 |

| | | | | |
|---|---|---|---|---|
| Parenteral nutrition on diagnosis, n (%) | 27 (20.5) | 12 (24.5) | 15 (18.1) | 0.377 |
| Enteral nutrition on diagnosis, n (%) | 125 (94.7) | 47 (95.9) | 78 (94) | 0.630 |
| **Outcome** | | | | |
| Death 28d, n (%) | 36 (27.3) | 17 (34.7) | 19 (22.9) | 0.141 |
| Death 90d, n (%) | 42 (31.8) | 20 (40.8) | 22 (26.5) | 0.088 |
| **Medication** | | | | |
| Immunosuppressants, n (%) | 33 (25) | 11 (22.4) | 22 (26.5) | 0.603 |
| Steroids > 10mg/day, n (%) | 28 (21.2) | 11 (22.4) | 17 (20.5) | 0.789 |
